# Array-based comparative genomic hybridization is more informative than conventional karyotyping and fluorescence in situ hybridization in the analysis of first-trimester spontaneous abortion

**DOI:** 10.1186/1755-8166-5-33

**Published:** 2012-07-16

**Authors:** Jinsong Gao, Congcong Liu, Fengxia Yao, Na Hao, Jing Zhou, Qian Zhou, Liang Zhang, Xinyan Liu, Xuming Bian, Juntao Liu

**Affiliations:** 1 Department of Obstetrics and Gynecology, Peking Union Medical College Hospital, Chinese Academy of Medical Science, Shuai Fu Yuan No.1, Dongdan, Beijing 100730, People's Republic of China; 2 Department of Molecular Genetics & Cytogenetics, Peking Union Medical College Hospital, Chinese Academy of Medical Science, Shuai Fu Yuan No.1, Dongdan, Beijing 100730, People's Republic of China; 3 BioChain (Beijing) Science and Technology Inc., No.7A, Yongchang North Rd, Beijing Economic-technological Development Area, Beijing 100176, People's Republic of China

**Keywords:** Spontaneous abortion, Aneuploidy, Karyotyping, Array-based comparative genomic hybridization

## Abstract

**Background:**

Array-based comparative genomic hybridization (aCGH) is a new technique for detecting submicroscopic deletions and duplications, and can overcome many of the limitations associated with classic cytogenetic analysis. However, its clinical use in spontaneous abortion needs comprehensive evaluation. We used aCGH to investigate chromosomal imbalances in 100 spontaneous abortions and compared the results with G-banding karyotyping and fluorescence *in situ* hybridization (FISH). Inconsistent results were verified by quantitative fluorescence PCR.

**Results:**

Abnormalities were detected in 61 cases. aCGH achieved the highest detection rate (93.4%, 57/61) compared with traditional karyotyping (77%, 47/61) and FISH analysis (68.9%, 42/61). aCGH identified all chromosome abnormalities reported by traditional karyotyping and interphase FISH analysis, with the exception of four triploids. It also detected three additional aneuploidy cases in 37 specimens with ‘normal’ karyotypes, one mosaicism and 10 abnormalities in 14 specimens that failed to grow *in vitro*.

**Conclusions:**

aCGH analysis circumvents many limitations in traditional karyotyping or FISH. The accuracy and efficiency of aCGH in spontaneous abortions highlights its clinical usefulness for the future. As aborted tissues have the potential to be contaminated with maternal cells, the threshold value of detection in aCGH should be lowered to avoid false negatives.

## Background

Spontaneous abortion is a common clinical problem that affects 10–15% of all clinically recognized human pregnancies, and mostly occurs in the first trimester [1]. Unbalanced chromosomal abnormalities account for 50–60% of fetal loss during this period, based on the results of cytogenetic studies [2] that provide valuable insights into the possible genetic causes of miscarriage and can determine recurrent risks.

Classic cytogenetic analysis is often the only genetic laboratory evaluation performed for spontaneous abortions. However, it has many limitations in the analysis of miscarriage. It relies on the successful culture of fetal tissue and preparation of metaphase cells, yet the successful rate of conventional karyotyping of miscarriage tissue is relatively low, ranging from 60 to 90% because of the *in vivo* death of tissue associated with spontaneous abortion, technical problems with culture growth or poor chromosome morphology [3-5]. Moreover, the information it provides is limited to numerical chromosomal abnormalities and gross structural rearrangements at a resolution of 5–10 Mb [6]. It has also been suggested that classic cytogenetics of spontaneous abortion might yield a false-positive normal karyotype or selected abnormal karyotype that permits cell proliferation *in vitro*[7]. In addition, standard cytogenetic diagnosis is a labor-intensive procedure, requiring the short- or long-term culture of fetal tissue.

Other rapid molecular cytogenetic techniques such as fluorescence *in situ* hybridization (FISH), quantitative fluorescence polymerase chain reaction (QF-PCR) and subtelomeric multiplex ligation-dependent probe amplification, which do not require cell culture, avoid some of these karyotyping pitfalls [8,9]. However, although these techniques detect the majority of chromosomal aberrations in spontaneous miscarriages, they use probes and primers that only target a selection of chromosomes or specific subtelomeric loci, thereby missing information about the remaining genome [10-12].

Array-based comparative genomic hybridization (aCGH) is a powerful new molecular cytogenetic technique that enables the genome-wide analysis of DNA copy numbers. It allows the simultaneous screening of gains and losses at thousands of targets while offering the advantages of high resolution and high throughput [13]. Many of the limitations of routine G-banding analysis, including cell culture failure and poor chromosome morphology, are circumvented by the use of genomic DNA. Unbalanced gains or losses of genetic material across the genome including those invisible to G-banding analysis can also be detected by aCGH [14-16].

When aCGH techniques are employed in the postnatal and prenatal population, there appears to be an increased detection rate of chromosomal imbalances, compared with conventional karyotyping [14,17,18]. Recently, aCGH has been considered a particularly useful alternative to conventional karyotyping in the field of diagnosis. However, although it is rapidly becoming the primary tool for the postnatal genetic evaluation of neonates and infants with dysmorphic features or cognitive difficulties, its use in routine prenatal diagnosis and miscarriage evaluation is still being investigated.

Thus far, the application of aCGH in miscarriages has been limited to no more than 500 cases worldwide. To our knowledge, seven studies have evaluated aCGH in the analysis of spontaneous abortion specimens [7,19-24]. aCGH appears to have an increased detection rate of chromosomal abnormality compared with conventional karyotyping. It was capable of detecting additional abnormalities in about 10% cases with normal karyotype mainly due to maternal cell contamination or submicroscopic chromosomal changes, and nearly 50% abnormalities in samples with culture failure when karyotyping was impossible. As it has also been suggested that lethal submicroscopic chromosomal changes can cause miscarriages [25,26], aCGH could become a complementary or even alternative method to traditional cytogenetic technique.

There are nearly 20,000,000 neonates each year in China with 10–15% ending in miscarriages. However, the genetic analysis of spontaneous abortions is seldom carried out because of limited cytogenetic resources. For this reason, the application of aCGH in spontaneous abortion analysis has never been reported in Chinese populations, yet it needs further evaluation for its clinical use in the detection of genomic imbalances in the cytogenetic evaluation of spontaneous miscarriages.

In this study, therefore, 100 spontaneous abortion specimens analyzed by G-banding and multiplex FISH were tested by aCGH arrays to evaluate the accuracy and efficiency of aCGH in the analysis of first trimester spontaneous abortions.

## Results

**Figure 1:**
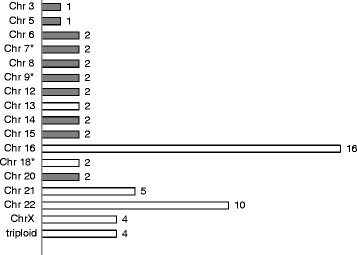
**Chromosome distribution of 61 abnormalities within (white column, 43 cases) or beyond (black column, 18 cases) FISH probe sets.** Approximately 30% of abnormalities could not be detected using these seven FISH probes. Most chromosome abnormalities were aneuploids, with the exception of four triploids and three segmental imbalances. *Segmental imbalances included one del 7p21.3-22.3, one dup 9p and one del 18p. The del 18p was not detected by FISH analysis as the probe was targeted at the centromere of chromosome 18. Chr: Chromosome.

Karyotyping failed in 14 (14%) samples as a result of culture failure, while aCGH and FISH analyses were successful in all cases. Overall, chromosome abnormalities were detected in 61 (61%) of the 100 spontaneous abortion specimens. Culturing detected abnormalities in 47 of all specimens (54.6% of those successfully karyotyped, and 77% of all abnormalities). FISH analysis using three probe sets targeting chromosomes 13, 16, 18, 21, 22, X and Y detected 42 abnormalities (accounting for 68.9% of all abnormalities). The remaining 19 chromosomal aberrations (31.1%) involving 11 different chromosomes were not detected by FISH because of probe limitations. aCGH detected chromosomal aberrations in 57 samples (93.4% of all abnormalities) but missed four triploid cases. The chromosome distribution of abnormalities is shown in Figure 1.

In accordance with previous reports, autosomal trisomy was predominant, accounting for 80.3% (49/61) of all chromosome abnormalities. Trisomy 16 (n = 16) and 22 (n = 10) were the most frequently encountered abnormalities, while trisomy 21 (n = 4), 45, X (n = 4) and triploid (n = 4) were the joint second most common abnormalities. These account for 62.3% (38/61) of all abnormalities.

A comparison of conventional cytogenetic, FISH and aCGH analyses is shown in Table 1. Seventy-five cases (87.2% of those successfully karyotyped) analyzed by aCGH were in exact concordance with the karyotype results. Of these matches, 32 showed a normal karyotype and 43 showed abnormal results in both tests, including 39 cases with autosomal trisomy, two cases with sex chromosome abnormalities (monosomy X), one case with monosomy 21 and one case with an 18p deletion.

**Table 1:** Comparison of karyotyping, FISH and aCGH analysis (n = 86)

**Case No**	**Karyotype results**	**FISH results**	**Array CGH results**^**e**^
1–11	46, XX	XX	Normal
12–13^a^	46, XX	XY	Normal
14^a^	46, XX	XY	+5^b^
15–35	46, XY	XY	Normal
36	46, XY	XY, +21	+21
37	46, XY	XY	+14^b^
38	47, XX, +3	XX	+3
39	47, XX, +6	XX	+6
40	47, XY, +6	XY	+6
41	47, XX, +7	XX	+7
42	47, XX, +8	XX	+8
43	47, XX, +9	XX	+9
44–45	47, XY, +12	XY	+12
46	47, XX, +13	XX, +13	+13
47	47, XX, +14	XX	+14
48	47, XX, +15	XX	+15
49-53	47, XX, +16	XX, +16	+16
54-62	47, XY, +16	XY, +16	+16
63	47, XY, +16	XY, +16/XY mosaic	+16
64	47, XX, +18	XX, +18	+18
65-66	47,XX,+20	XX	+20
67-68	47,XX,+21	XX, +21	+21
69	47,XY,+21	XY, +21	+21
70-72	47,XX,+22	XX, +22	+22
73	47,XX,+22	XX,+22/XX mosaic	+22
74	47,XX,+22	XX, +22	+9 ^b^ ,+22
75-77	47,XY,+22	XY, +22	+22
78	46,XY,del 18p	XY	del 18p
79	45,XY,-21	XY, -21	−21
80-81	45,X	Monosomy X	Monosomy X
82-84	Triploid	Triploid	Normal
85-86	Tetraploid ^c^	Normal	Normal
All abnormal	47^d^	35	47

^a^only maternal cell cultured.

^b^verified by QF-PCR.

^c^due to a culture artifact (92,XXXX and 92,XXYY).

^d^excluding two culture artifact tetraploids.

^e^The sex chromosome in aCGH was in accordance with FISH analysis.

**Figure 2:**
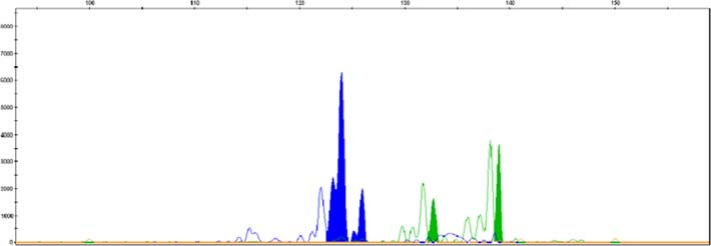
**Electrophoretograms of QF-PCR amplifications in case 37.** The x-axis shows the length of the PCR products in base pairs and the y-axis shows the fluorescence intensity in arbitrary units. DNA samples were amplified with two markers for chromosome 14: D14S985 (blue) and D14S1007 (green). Both markers are informative and show two peaks with a 2:1 ratio, confirming the result of trisomy 14 in array CGH.

Discrepancies between aCGH and cytogenetic results occurred in 11 (12.8%) of 86 karyotyped cases (Table 1). Three 46, XX karyotypes (cases 12–14) were proved to be a maternal overgrowth of fetal cells, with one (case 14) diagnosed as a trisomy 5 male by aCGH analysis. Two 46, XY karyotypes were diagnosed as trisomy 21 (case 36) and trisomy 14 (case 37) by aCGH. One trisomy 22 shown by karyotype and FISH analysis (case 74) was suggested instead to be a mosaicism for trisomy 22 and trisomy 9 by aCGH analysis. The additional chromosome abnormalities detected by aCGH were all verified by FISH analysis or QF-PCR (Figure 2). Three triploids diagnosed by karyotype and FISH analysis (cases 82–84) were all found to be normal by aCGH. Two tetraploid cases (85 and 86) were proved to be culture artifacts. One trisomy 16 was confused with trisomy 20 because of poor chromosome morphology. It was later confirmed to be trisomy 16 by aCGH.

In 14 specimens that failed to yield a karyotype, 11 (78.6%) showed abnormalities as detected by either FISH or aCGH. aCGH results were then compared with FISH analysis (Table 2). FISH analysis verified six aCGH results but missed one trisomy 8, one trisomy 15, one 9p duplication and one 7p deletion (del 7p21.3-p22.3,-11.72 Mb), which were not identifiable by the probe sets. One normal sample in aCGH was proven to be triploid by FISH analysis.

**Table 2:** Comparison of FISH and aCGH analysis of cases without karyotype results (n = 14)

**Case No.**	**FISH**	**aCGH**^**a**^
87	XX	Normal
88	XY	Normal
89	XY	Normal
90	XX	del 7p21.3-p22.3 (−11.72 Mb)
91	XX	+8
92	XX	dup 9p
93	XX	+15
94	XY, +13	+13
95	XY, +16	+16
96	XX, +22	+22
97	XY, +22	+22
98	Monosomy X	Monosomy X
99	Monosomy X	Monosomy X
100	Triploid	Normal
All abnormal	7	10

^a^The sex chromosome in aCGH was in accordance with FISH analysis.

Out of 61 abnormal specimens, we detected seven samples with a high maternal cell contamination (MCC) rate by reducing the threshold value of fold change in aCGH. Table 3 gives the MCC rates of these specimens.

**Table 3:** Abnormal samples with high MCC rate detected by lower threshold value of aCGH

**Case No.**	**Routine threshold value** **Log**_**2**_**ratio = 0.38**	**Lowered threshold value** **Log**_**2**_**ratio = 0.26**	**MCC rate (%)**
40	Normal	+6	17.1
41	Normal	+7	30.8
53	Normal	+16	29.2
59	Normal	+16	22.3
60	Normal	+16	20.6
61	Normal	+16	18.8
62	Normal	+16	22.3

MCC: maternal cell contamination.

## Discussion

In this study, aCGH was compared with traditional karyotyping and interphase FISH analysis for the detection of chromosomal abnormalities in spontaneous abortions. Overall, aCGH showed the highest detection rate (93.4%, 57/61) compared with karyotyping (77%, 47/61) and FISH analysis (68.9%, 42/61). Our study demonstrates that aCGH analysis of spontaneous abortions is both accurate and efficient, which augurs well for its clinical usefulness. To our knowledge, this is the first such study to be reported of the Chinese population.

The distribution of chromosome abnormalities identified in our study was similar to other reports [6,12] (see Figure 1). However, submicroscopic imbalances were less prevalent, most likely because they were not lethal. We identified one case with a single copy loss of 11.72 Mb in the 7p21.3-p22.3 region. Such deletions have previously been found in postnatal patients with mental retardation or development delay [27], suggesting that the spontaneous abortions caused by this microdeletion must be occasional even if they are considered pathogenic.

In another case, the array data suggested mosaicism for trisomy 9 and 22, which was not identified by cytogenetic analysis. It is conceivable that the cell line with trisomy 9 is slow to grow and divide, thereby presenting very few mitotic cells. This example highlights the limitation of cytogenetic analysis which relies on cell culture and demonstrates the potential of aCGH to detect the presence of mosaicism [22].

Karyotyping inaccuracies have been shown to be a result of maternal cell overgrowth or selected karyotypes that permit cell proliferation *in vitro *[7]. The culture failure rate and MCC rate of our study were similar to those of other reports [3,17,28], although the cytogenetic factors of cell death *in vitro* have not been thoroughly investigated. It is possible that cell culture failure is a marker of particular genomic imbalances incompatible with normal cell proliferation. As shown in our results, abnormalities were more frequently detected in specimens with culture failure. If this hypothesis is true, then the standard cytogenetic analysis of spontaneous abortions may underestimate the frequency and diversity of detected chromosomal abnormalities.

Insufficient enumeration probes could cause inaccuracies in FISH analysis. As shown in Figure 1, the distribution of aberration was scattered throughout most chromosome types. Although approximately 70% chromosome abnormalities could be detected by this multiplex interphase FISH, consistent with other reports [12], more than 30% of abnormalities could not be detected. As the use of probes that target all chromosomes would be expensive and unrealistic, this limits the application of FISH in the complete analysis of spontaneous abortion.

Aborted tissue is a mixture of conceptus, chorionic villi and deciduas, so is likely to be contaminated with maternal cells. It is therefore essential to carefully detach the deciduas from the chorionic villi as much as possible to decrease the MCC rate, although this is difficult to achieve. To overcome this problem, we reduced the detection threshold to a 1.2-fold change (0.26 log_2_ ratio) to see if this would increase the detection rate without increasing the false positive rate. Under this threshold value, we detected seven additional chromosomal aberrations that had originally showed a negative result under aCGH routine protocols. Thus it seems reasonable to adjust the threshold value to 0.26 log_2_ ratio fold change when analyzing spontaneous abortion specimens with aCGH. Although all deviations from the normal range were considered to be MCC, placenta mosaicisms would also meet this analysis criterion. However, distinguishing between these two situations has no obvious clinical significances. In addition, different microarray platforms have their own system noises. Since we deduce the W value and calculate the MCC rate (or mosaic rate) on an Agilent 60-mer Oligo aCGH platform (see Methods), it is important that the W value and its standard error in this algorithm be Agilent platform-specific.

A limitation of aCGH in the analysis of spontaneous abortion is that it cannot reliably detect polyploidy, which accounts for about 2–10% of all spontaneous abortions [10,28]. This is mainly because of the global normalization used in microarray-based methods. Although a previous study overcame these problems by using 47,XXY cells as control DNA to enable the detection of some triploids [29], this was not conducted in the present study but remains a possibility for future work.

## Conclusions

The present study demonstrates that the DNA-based aCGH technology overcomes many limitations of routine cytogenetic analysis used in the analysis of spontaneous abortion specimens while enhancing the detection rate of chromosome aberrations. Although its current costs are relatively expensive, we expect the price to decrease in the near future with increases in the number of arrays consumed in clinical applications. At that time, aCGH may become a cost-effective method in the analysis of spontaneous abortion and other chromosome abnormality diagnosis.

## Methods

### Specimen preparation

A total of 100 samples were received from women who experienced spontaneous miscarriage before 12 weeks of gestation at Peking Union Medical College Hospital, Beijing, China. All patients gave informed consent for participation in the research. The mean maternal age of the patient group was 32 years (range, 24–45 years); all were primigravidae, although there had been a previous abortion in two cases.

After termination of the pregnancies, a small portion of chorionic villi samples (CVS) was placed in a sterile container with 0.9% normal saline and sent to the laboratory within 24 hours. The specimen was examined grossly, washed clean of blood and maternal deciduas and divided into three parts for cell culture, preparation of noncultured single-cell suspensions and DNA extraction.

### Karyotype analysis

Approximately 20–30 mg CVS tissue was used for cell culture. Following collagenase and trypsin dissociation of the specimen, two primary cultures from the tissue samples were established using standard methods. Cultures were harvested after approximately 1–2 weeks. Karyotyping was performed on G-banded chromosome preparations. A minimum of five, but preferentially 20, cells were analyzed per sample.

### FISH analysis

About 5 mg of tissues was used for FISH analysis. Noncultured single-cell suspensions were prepared by disaggregation in 60% acetic acid, then fixed and stored in 3:1 methanol/acetic acid.

Based on knowledge about the frequencies of specific trisomies in spontaneous abortions and the availability of commercial multiplex probe sets, FISH was performed using three multicolor probe mixtures (GP Medical Technologies Inc., Beijing, China). Probe mix 1 and mix 2 contain locus-specific probes that identify chromosomes 13, 21 and 16, 22 respectively. Probe mix 3 contains centromere site probes that identify chromosomes 18, X and Y. Each probe set was applied to one of the two slides from each case. The probes were direct-labeled with different fluorophores, which could be visualized with appropriate filter combinations. Hybridization and post-washing conditions were performed according to the manufacturer’s instructions. Slides were counterstained with 15 ml of a very dilute 4,6-diamidino-2-phenylindole antifade solution for 10 minutes and observed under a fluorescent microscope (BX51, Olympus, Tokyo, Japan) equipped with an appropriate filter.

Nuclear signals were scored using a × 100 oil objective. Fifty nuclei were scored for each of the seven chromosomes. The cut-off rates scheme was prepared according to a previous report [30] and our own experience. In normal diploid cells, two signals were observed for each chromosome from a mean of 90% of scored cells. Aneuploidy was diagnosed when >60% of cells showed an abnormal number of signals. More nuclei (≥100) were scored if 10–60% of cells showed abnormalities. A value between 10 and 20% was regarded as unclear but likely to be normal. A value >20% was regarded as mosaicism or suspected aneuploidy.

### aCGH analysis

aCGH analysis was used to examine all 100 spontaneous abortion samples. Total DNA was extracted from uncultured CVS tissues with a commercially available Genomic DNA Extraction Kit (BioChain Institute Inc., Newark, CA) according to the manufacturer's instructions. For each aCGH experiment, 400 ng each of purified DNA and normal sex-matched DNA (BioChain Institute) was digested with 10 U *Alu* I and 10 U *Rsa* I (Promega, Madison, WI) and differentially labeled with cyanine-5 (cy5) and cyanine-3 (Cy3) fluorescent dyes using a Genomic DNA Enzymatic Labeling Kit (Agilent, Santa Clara, CA). aCGH analysis was performed using 8 × 60 K commercial arrays (Agilent). This platform contains 60-mer oligonucleotide probes spanning the entire human genome with an overall mean probe spacing of 50 kb. After hybridization, the arrays were scanned using a dual-laser scanner (Agilent) and the images were extracted and analyzed using Feature Extraction software (Agilent) and Workbench genomics software, respectively.

### MCC analysis

An aborted conceptus is likely to be contaminated with maternal cells, resulting in the failure of karyotyping analysis. In aCGH analysis, DNA was directly extracted from CVS tissues so might be expected to contain maternal DNA. If MCC-containing CVS samples are analyzed according to unitary samples, false negative CVS DNA abnormalities may be revealed. We therefore adopted a mixing algorithm to detect the chromosome abnormalities in CVS tissues. Since trisomies account for most spontaneous abortions, we used trisomy 21 as an example to establish the mixing model. Thus, in a trisomy sample contaminated with maternal cells, the measured ratio is M = R + (1-R)*T. This algorithm has been used in the aCGH analysis of tumor tissues, which often contain normal cells [31]. Here, R is the MCC proportion, and T is the ratio of trisomy DNA to normal control, which should theoretically equal 1.5, inferring that M = 1.5-0.5R. The M value is linear to R. When R equals zero, representing no contamination, the M value will be 1.5; when R equals 1.0, meaning that all cells derive from the mother, the M value will also be 1.0.

To detect chromosome abnormalities, a threshold value should be determined considering the true biological change and the system noise. Agilent aCGH standard protocols set the threshold value as a 1.3-fold change for one copy number amplification or loss, which corresponds to a Log_2_ ratio < −0.38 or >0.38. When considering MCC in miscarriage tissues, we established an equation M = Mc + W, where Mc denotes the threshold value of fold change that optimally detects trisomy. Above the M_c_ value no abnormality is detected, while below the M_c_, the abnormality is reported. W is a constant reflecting precision of the aCGH platform that can be estimated by comparing the non-contaminated trisomy samples with normal samples. We employed 10 trisomy 21 syndrome samples from peripheral blood and calculated W = 0.11 ± 0.04. Deducing from M = Mc + W for Mc = 1.39, this suggests that the trisomy DNA is not contaminated with normal DNA, which agrees with a previous comparison between three and two copies of the X chromosome [32].

To detect chromosome abnormalities in miscarriages with potential MCC, we reduced the detection threshold to a 1.2-fold change (0.26 log_2_ ratio) with at least 10 consecutive probes. Using this threshold value, the MCC rate was 32.5%. Reducing the threshold still further could lead to the emergence of false positives because of platform system noise (data not shown).

### QF-PCR

QF-PCR of DNA was performed to verify the results of aCGH if these revealed unbalanced chromosomal abnormalities that did not correlate with the karyotype. By amplifying highly polymorphic regions of short tandem repeats (STR) specific for a particular chromosome, we could detect dosage ratios of the PCR products by analyzing the fluorescent peak areas shown by a Genetic Analyzer. In normal heterozygotes, the ratio of fluorescent activity for the two peaks corresponding to the PCR products should be within the range 0.8–1.4 (disomic diallelic). In a trisomic specimen, the three doses of an STR marker can be detected either as three peaks of fluorescent activities with a 1:1:1 ratio (trisomic triallelic) or as a pattern of two peaks with a 1:2 ratio (trisomic diallelic) [33].

## Competing interest statement

The authors declare that they have no competing interests.

## Authors’ contributions

JG drafted the manuscript, performed array CGH and carried out data analysis. CL performed FISH. FY performed QF-PCR. NH and JZ performed karyotyping. QZ and LZ performed array CGH and participated in the study coordination. XL performed clinical evaluations of the pregnancies and participated in the coordination of the project. XB performed clinical evaluations of the pregnancies and helped to draft the manuscript. JL conceived the study, participated in its design and coordination and also approved the manuscript. All authors have read and approved the manuscript.
